# Synchronous Extensive-Stage Small Cell Lung Cancer and Multiple Myeloma Detected by Marked Hypergammaglobulinemia

**DOI:** 10.7759/cureus.103798

**Published:** 2026-02-17

**Authors:** Makoto Fujimoto, Toyoshi Yanagihara, Hiroki Ueno, Mikiko Aoki, Makoto Hamasaki, Yasushi Isobe, Noriyuki Ebi, Hiroyuki Inoue, Masaki Fujita

**Affiliations:** 1 Department of Respiratory Medicine, Fukuoka University Hospital, Fukuoka, JPN; 2 Department of Pathology, Fukuoka University Hospital, Fukuoka, JPN; 3 Department of Pathology, Fukuoka University, Fukuoka, JPN; 4 Department of Internal Medicine, Division of Medical Oncology, Hematology, and Infectious Disease, Fukuoka University Hospital, Fukuoka, JPN

**Keywords:** carcinomatous pleuritis, diagnosis of multiple myeloma, extensive stage small cell lung cancer, igg hypergammaglobulinemia, synchronous cancers

## Abstract

A 75-year-old woman presented with nausea, exertional dyspnea, and right-sided chest pain. Chest imaging showed a right hilar mass with right pleural effusion. Initial assessment favored primary lung cancer with carcinomatous pleuritis. However, laboratory tests at admission revealed markedly elevated levels of total protein with hypoalbuminemia, resulting from monoclonal IgG-kappa (κ) gammopathy (IgG: 7,700 mg/dL). Thoracentesis demonstrated an exudative effusion; pleural fluid cytology and transbronchial tumor biopsy confirmed small cell lung cancer (SCLC). Anemia, markedly increased serum free κ light chain levels, and atypical plasmacytosis in the bone marrow confirmed the presence of multiple myeloma. She was then diagnosed with extensive-stage SCLC and concomitant multiple myeloma. Dose-reduced carboplatin plus etoposide was initiated for SCLC with clinical improvement and no severe acute toxicity; treatment for myeloma was planned after stabilization of SCLC. This case highlights that extreme hypergammaglobulinemia in a patient with suspected lung cancer should trigger prompt evaluation for multiple myeloma. Dual malignancy can coexist and may be overlooked if clinicians focus on a single diagnosis.

## Introduction

Small cell lung cancer (SCLC) is an aggressive neuroendocrine carcinoma that often presents with a central/hilar tumor and pleural effusion. When pleural effusion is present, cytology and cell block immunohistochemistry are central to establishing the diagnosis and guiding management. Multiple myeloma (MM) is a clonal plasma cell disorder characterized by monoclonal immunoglobulin production and the presence of end-organ damage, including hypercalcemia, renal failure, anemia, and bone lesions. Synchronous presentation of SCLC and MM is extremely rare [1]. Most reports of coexisting lung cancer and MM involve non-small cell lung cancer or metachronous disease [2,3]. Synchronous SCLC and MM are also challenging to detect because pleural effusion and systemic findings, such as anemia or fatigue, may be attributed to SCLC, leading to diagnostic anchoring and delayed evaluation for a second malignancy. Here, we report a patient in whom an unusually high total protein-to-albumin dissociation led to the recognition of elevated levels of serum monoclonal IgG-kappa (κ) and ultimately to the diagnosis of synchronous extensive-stage SCLC and MM.

## Case presentation

**Figure 1:**
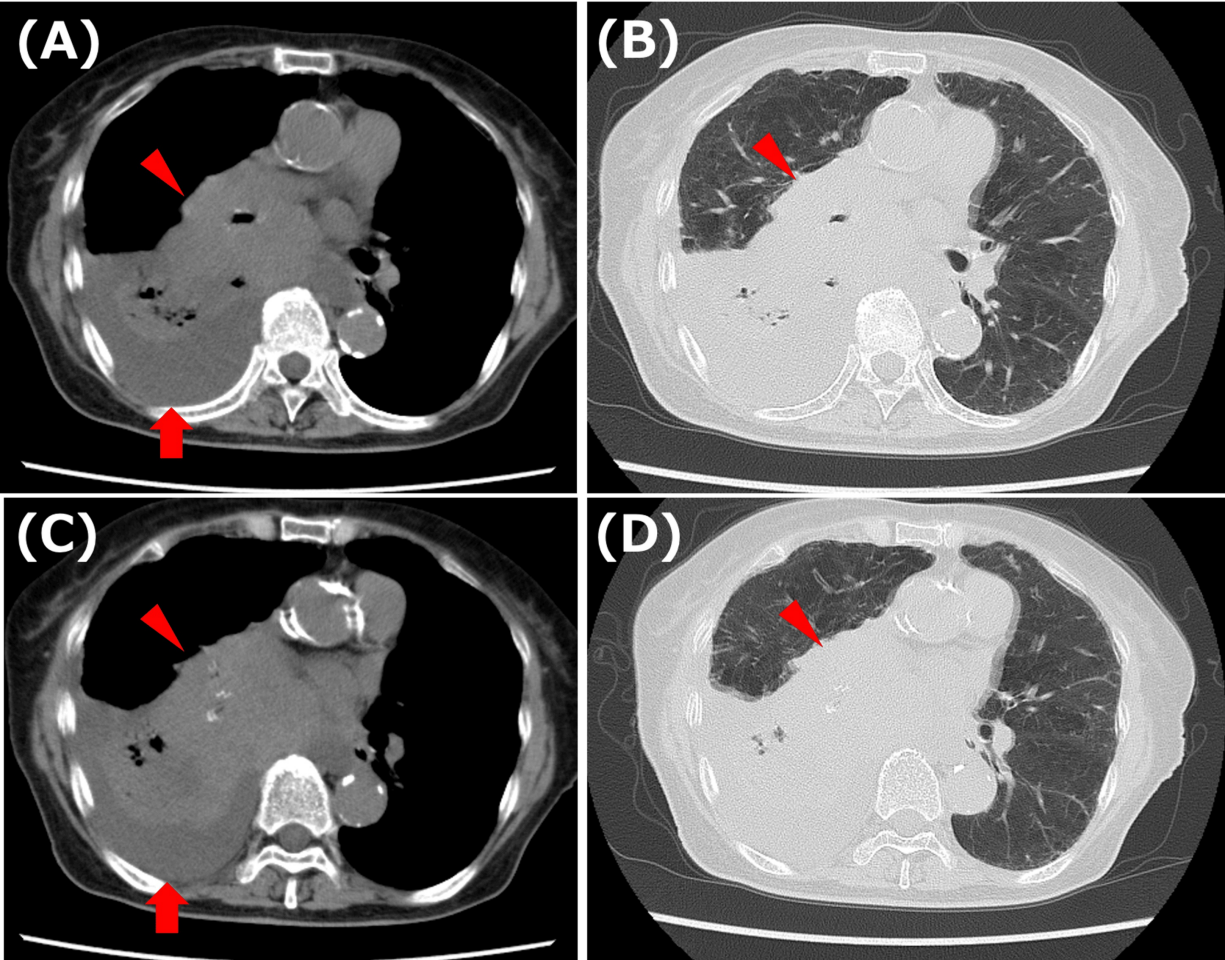
Chest CT findings on admission. Axial CT images in the mediastinal window (A, C) and corresponding lung-window (B, D) images demonstrate a right hilar mass (arrowheads) with a right pleural effusion (arrows).

A 75-year-old woman presented with nausea, exertional dyspnea, and right-sided chest pain. Two months before admission, she had been hospitalized for a sacral fracture and improved with conservative management. After discharge, she developed persistent nausea and dyspnea on walking, along with cough-associated right chest pain, and was referred for evaluation. Chest CT revealed a right hilar mass and pleural effusion, and she was admitted for further workup (Figures 1A-1D). Past medical history included hypertension. Medications included amlodipine 5 mg. She had a substantial smoking history (15-20 cigarettes/day for 50 years).

**Figure 2:**
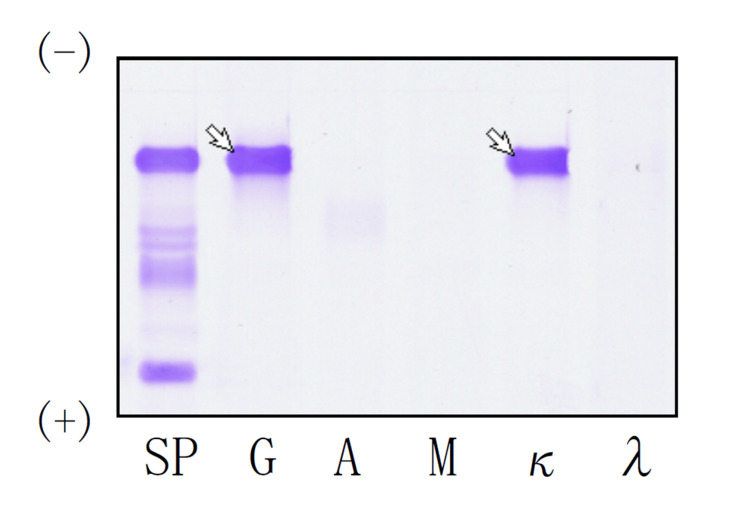
Serum immunofixation electrophoresis. Arrows indicate a discrete monoclonal band detected in the IgG (G) and κ lanes at the same electrophoretic mobility, consistent with an IgGκ monoclonal protein. SP: serum protein electrophoresis

On admission, vital signs were stable aside from hypertension (BP: 162/119 mmHg). Oxygen saturation was 97% on room air. Physical examination showed decreased breath sounds at the right lower lung field without crackles or wheezes and no peripheral edema. The initial working diagnosis was primary lung cancer with malignant pleural effusion. However, laboratory testing showed markedly elevated levels of total protein (TP) (10.9 g/dL) with hypoalbuminemia (2.5 g/dL), prompting further evaluation for hypergammaglobulinemia (Table 1). Serum immunoglobulin (Ig) quantification demonstrated a markedly elevated IgG level (7,268 mg/dL). Serum protein electrophoresis indicated a monoclonal spike, and immunofixation identified an IgGκ monoclonal protein (Figure 2). The serum free light chain assay further showed markedly increased levels of free κ chains (241 mg/L) and an elevated κ/λ ratio (20.44), indicating the presence of B-cell clonality. Serum pro-gastrin-releasing peptide (ProGRP) level was markedly elevated at 4,320 pg/mL.

**Table 1:** Laboratory findings on admission. Baseline hematology, biochemistry, immunoglobulin profile, tumor markers, and serum free light chain results obtained at presentation. WBC: white blood cell count; RBC: red blood cell count; Hb: hemoglobin; Plt: platelet count; TP: total protein; Alb: albumin; CRP: C-reactive protein; AST: aspartate aminotransferase; ALT: alanine aminotransferase; LDH: lactate dehydrogenase; BUN: blood urea nitrogen; Cr: creatinine; Na: sodium; K: potassium; UA: uric acid; Ca: calcium; IgG: immunoglobulin G; IgA: immunoglobulin A; IgM: immunoglobulin M; IgE: immunoglobulin E; IgG4: immunoglobulin G4; sIL-2R: soluble interleukin-2 receptor; CEA: carcinoembryonic antigen; CYFRA: cytokeratin 19 fragment (CYFRA 21-1); ProGRP: pro-gastrin-releasing peptide; κ: kappa; λ: lambda

Test	Value	Reference range
WBC (per μL)	7,400	3,300-8,600
RBC (10^4^/μL)	313	435-555
Hb (g/dL)	10	13.7-16.8
Plt (10^3^/μL)	210	158-348
TP (g/dL)	10.9	6.6-8.1
Alb (g/dL)	2.5	3.8-5.3
CRP (mg/dL)	1.12	0.4-1.5
AST (U/L)	29	13-30
ALT (U/L)	14	10-42
LDH (U/L)	265	142-222
BUN (mg/dL)	32	8-20
Cr (mg/dL)	0.86	0.65-1.07
Na (mEq/L)	139	138-145
K (mEq/L)	4.7	3.6-4.8
Ca (mg/dL)	8.4	8.8-10.4
UA (mg/dL)	6.2	2.6-5.5
IgG (mg/dL)	7,268	861-1747
IgA (mg/dL)	69	93-393
IgM (mg/dL)	24	50-269
IgE (IU/mL)	25	<232
IgG4 (mg/dL)	7	11-121
sIL-2R (U/mL)	730	121-613
CEA (ng/mL)	1.7	<5.0
CYFRA (ng/mL)	8.3	<3.5
ProGRP (pg/mL)	4,320	<81
Free κ chain (mg/L)	241	3.3-19.4
Free λ chain (mg/L)	11.8	5.7-26.3
κ/λ	20.44	0.26-1.65

**Figure 3:**
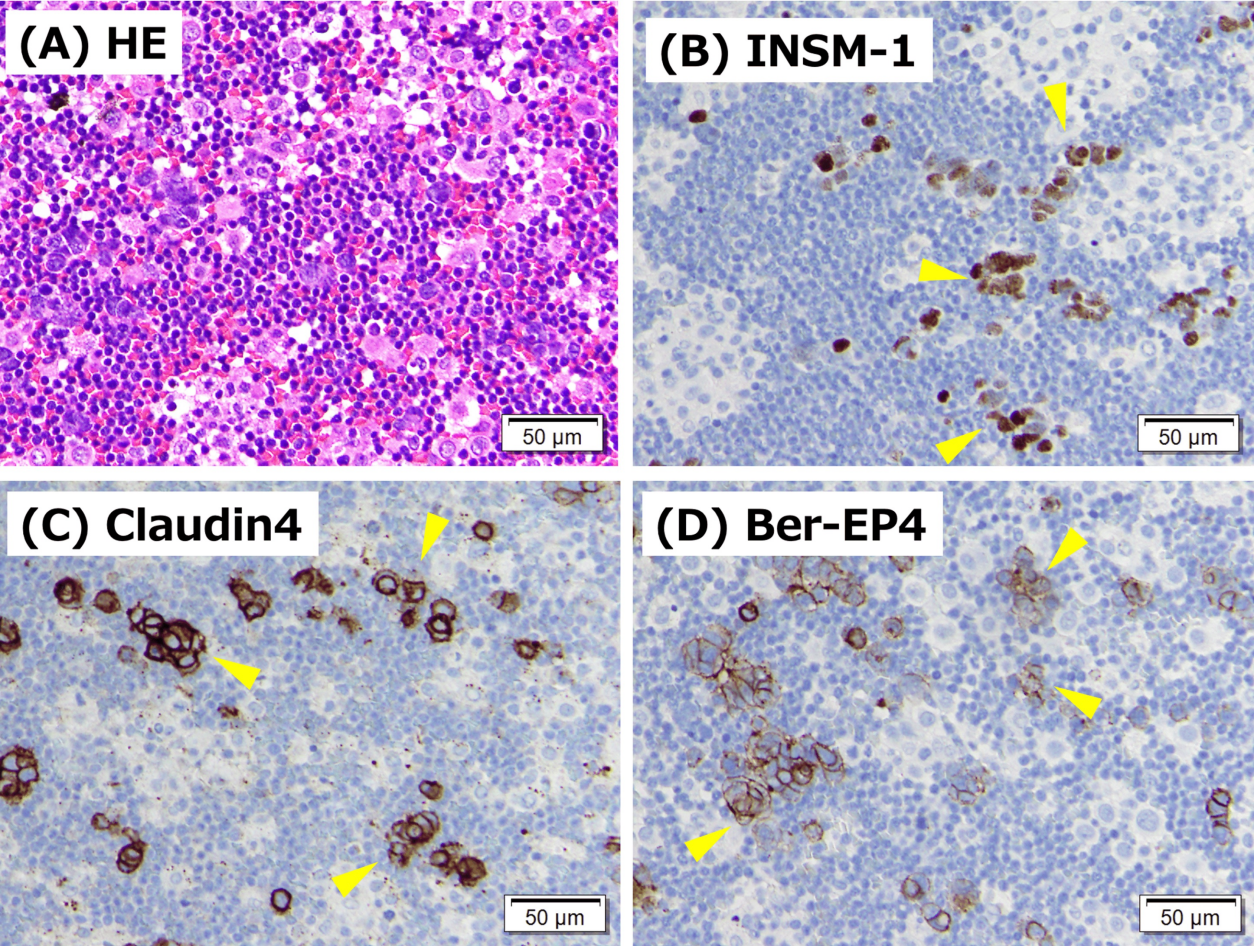
Pleural fluid cell block pathology on admission. (A) Hematoxylin and eosin staining shows clusters of small atypical cells with high nuclear-to-cytoplasmic ratios in the pleural effusion. Immunohistochemistry demonstrates positivity (arrowheads) for (B) insulinoma-associated protein 1 (INSM1), (C) Claudin-4, and (D) Ber-EP4, supporting a diagnosis of small cell lung carcinoma involving the pleural space. Scale bars, 50 μm.

**Figure 4:**
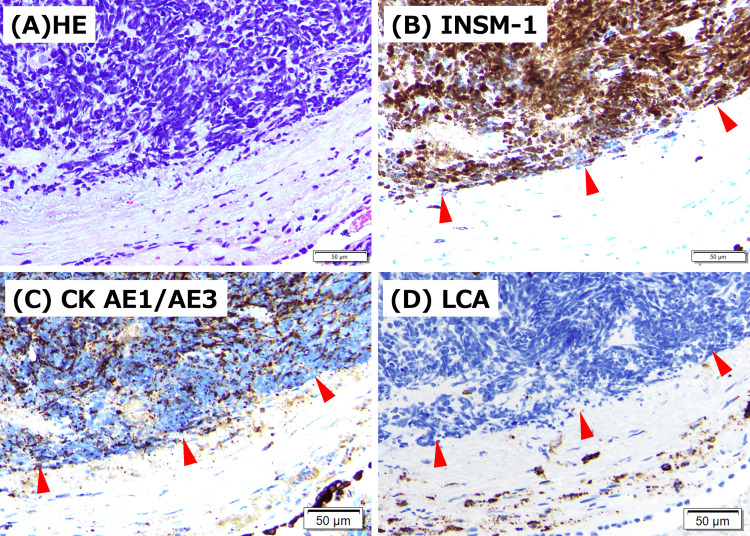
Transbronchial tumor biopsy findings. (A) Hematoxylin and eosin staining shows a proliferation of small atypical cells with scant cytoplasm and hyperchromatic nuclei. Immunohistochemistry demonstrates strong positivity (arrowheads) for (B) insulinoma-associated protein 1 (INSM1) and (C) CK AE1/AE3, supporting a diagnosis of small cell lung carcinoma. (D) LCA (leukocyte common antigen, CD45) is negative (arrowheads), arguing against lymphoma. Scale bars, 50 μm.

Right thoracentesis was performed. Cultures for bacteria and mycobacteria were negative. Pleural fluid satisfied Light’s criteria for an exudate. Cytology showed small atypical cells with increased chromatin and a high nuclear-to-cytoplasmic ratio, compatible with small cell carcinoma. On cell block immunohistochemistry, tumor cells expressed epithelial markers (Claudin-4, MOC-31, Ber-EP4) and neuroendocrine markers (CD56, synaptophysin, INSM-1), with TTF-1 also reported positive, supporting SCLC (Figures 3A-3D). Bronchoscopy with biopsy confirmed a neuroendocrine carcinoma profile, including TTF-1, cytokeratin (AE1/AE3), CD56, and INSM-1 positivity, LCA negative, consistent with SCLC and excluding lymphoma (Figures 4A-4D). Bone marrow examination revealed slightly hypoplastic marrow with atypical plasma cell proliferation comprising 42% of the bone marrow nucleated cells, consistent with multiple myeloma. Morphologically, the plasma cells were predominantly medium-to-large and appeared relatively immature, with prominent, enlarged Russell bodies. Therefore, she was diagnosed with extensive-stage SCLC and concomitant multiple myeloma. Brain magnetic resonance imaging (MRI) was attempted, but could not be completed due to the inability to remain still. Head CT showed no obvious brain metastasis. Systemic staging was performed with chest-abdomen CT, which did not demonstrate definite liver or adrenal metastases or obvious bone lesions. Whole-body positron emission tomography was not performed during this admission, which limits evaluation for occult metastases. For myeloma staging, serum albumin was 2.5 g/dL, and LDH was 265 U/L. Serum β2-microglobulin was not measured. Conventional karyotyping (G-banding) did not show chromosomal abnormalities. Fluorescence in situ hybridization (FISH)-based risk stratification was not available. Formal International Staging System (ISS) staging could not be completed, and interpretation of albumin and LDH was limited by the concomitant extensive-stage SCLC and acute illness.

**Figure 5:**
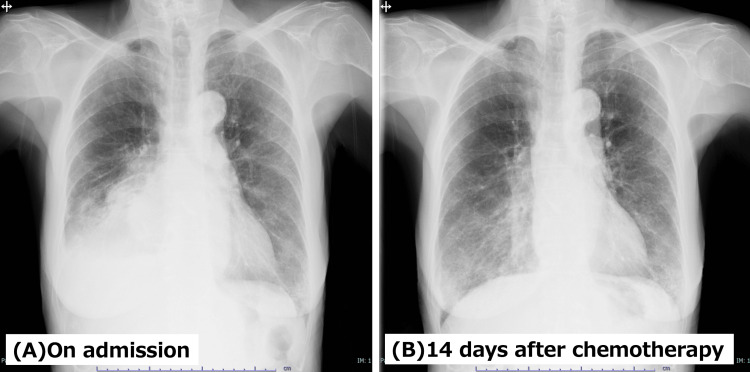
Serial chest radiographs before and after chemotherapy. (A) Chest radiograph on admission shows a large right pleural effusion with associated right basal atelectasis. (B) Chest radiograph 14 days after initiation of chemotherapy demonstrates a marked reduction in the right pleural effusion with improved right lung expansion.

After bronchoscopy, the patient began dose-reduced carboplatin plus etoposide as first-line therapy for SCLC. She had a favorable response to chemotherapy, and her general condition improved (Figures 5A, 5B). Treatment for multiple myeloma was deferred, with a plan to determine timing once SCLC disease control was achieved.

## Discussion

Synchronous occurrence of MM and lung cancer is rare, and the literature consists mainly of isolated case reports. Ji et al. described three Korean patients with synchronous MM and solid tumors, including a patient with synchronous SCLC and MM [1]. In more recent reports, coexisting MM and lung cancer are described predominantly with NSCLC [2,3]. These publications repeatedly emphasize that dual diagnoses are made only when routine laboratory abnormalities are interpreted together with confirmatory bone marrow studies and tumor histopathology. Our case is notable for extensive-stage SCLC with pleural effusion coexisting with MM, in which the initial clue to MM was a routine and immediately available laboratory pattern - markedly elevated total protein (10.9 g/dL) with low albumin (2.5 g/dL).

Marked dissociation between TP and albumin - often expressed as the protein (gamma) gap (TP-albumin) or as a low albumin-to-globulin ratio (A/G or AGR) - is a practical bedside clue to hypergammaglobulinemia and can serve as a trigger to evaluate for monoclonal gammopathy. For clinical use, commonly cited “red-flag” thresholds include a gamma gap of >4 g/dL (and, in some real-world analyses, approximately 4.6 g/dL) and/or a low A/G ratio. Importantly, available data indicate that these indices have limited sensitivity for monoclonal disorders and should be viewed as rule-in warning signs rather than rule-out tests. In a population-based analysis, a gamma gap threshold of >4 g/dL showed 15.4% sensitivity and 95.4% specificity for monoclonal gammopathy of undetermined significance (MGUS), indicating that many monoclonal cases do not meet this cutoff, whereas a positive result increases suspicion [4]. Consistent with this, a large real-world study evaluating screening performance for monoclonal proteins reported that a gamma gap cutoff of 4.6 g/dL had a sensitivity of 35% and a specificity of 91% [5]. In our patient, the gamma gap was 8.4 g/dL (TP: 10.9 g/dL; albumin: 2.5 g/dL), which prompted confirmatory testing. Marked TP-albumin dissociation can be caused by polyclonal hypergammaglobulinemia (chronic infection, autoimmune disease, or liver disease); however, the presence of IgGκ monoclonality with an abnormal κ/λ ratio and bone marrow plasmacytosis supported a plasma cell neoplasm rather than reactive hypergammaglobulinemia. Taken together, these findings support the clinical use of TP-albumin dissociation as an early warning signal that should prompt definitive testing - serum protein electrophoresis with immunofixation, serum free light chains, urine studies, and bone marrow examination.

The presenting combination of lung mass, pleural effusion, and high IgG can overlap with inflammatory and lymphoproliferative disorders, particularly IgG4-related lung/pleural disease and multicentric Castleman disease [6,7]. The key distinction is clonality. Assessment of κ/λ restriction and M-protein, supported by bone marrow evaluation, can rapidly separate clonal plasma cell neoplasms from reactive polyclonal plasmacytosis, thereby preventing misclassification of MM as an inflammatory mimic. In our case, the serum IgG4 level was low (7 mg/dL), making IgG4-related lung/pleural disease unlikely. Moreover, the diagnoses of MM and SCLC were confirmed by bone marrow pathology and histopathologic evaluation of the lung tumor.

Finally, coexisting SCLC and MM complicates therapeutic planning because treatment must be sequenced to control the immediately life-threatening process while maintaining hematologic reserve and limiting overlapping toxicities [1,8]. We prioritized SCLC-directed therapy because thoracic disease was clinically dominant, initiating dose-reduced carboplatin plus etoposide after bronchoscopy. The patient improved without severe acute toxicity, and MM-directed therapy was deferred for reassessment after stabilization of SCLC. When both malignancies require near-simultaneous treatment, careful coordination is required for myelosuppression, infection prevention, renal monitoring, transfusion support, thrombosis risk, and potential drug-drug interactions. Reporting practical sequencing decisions and toxicity considerations is valuable because synchronous SCLC and MM remains a rare scenario in routine practice. A limitation is that formal ISS staging was not available because β2-microglobulin and FISH were not obtained, and albumin/LDH were potentially confounded by concomitant extensive-stage SCLC (ES-SCLC).

## Conclusions

Synchronous SCLC and MM is rare, but clinically important because diagnostic anchoring to a single malignancy can delay recognition of the second malignancy. In our patient, extreme hypergammaglobulinemia with a marked TP-albumin dissociation was the pivotal clue that prompted prompt myeloma workup and confirmed dual pathology. When lung cancer is accompanied by markedly elevated IgG and pleural plasmacytosis, clinicians should actively evaluate for plasma cell neoplasms using clonality testing and bone marrow examination.
